# [μ-Bis(diphenyl­phosphan­yl)acetonitrile-κ^2^ *P*:*P*]bis­[chloridogold(I)]

**DOI:** 10.1107/S1600536810049834

**Published:** 2010-12-11

**Authors:** Sicelo V. Sithole, Richard J. Staples, Werner E. van Zyl

**Affiliations:** a School of Chemistry, University of KwaZulu-Natal, Westville Campus, Private Bag X54001, Durban 4000, South Africa; b Department of Chemistry, Michigan State University, East Lansing, MI 48824-1322, USA

## Abstract

The title complex, [Au_2_Cl_2_(C_26_H_21_NP_2_)], has an intra­molecular Au⋯Au inter­action of 3.1669 (4) Å, but no inter­molecular Au⋯Au inter­actions in the solid state. The Cl—Au—P bond angle of 176.84 (7)° is slightly distorted from linearity. The P—C bond length to the phenyl group is shorter [1.810 (7) Å] than the P—C bond length [1.876 (7) Å] to the bridging carbon, indicative of the flexibility of the bidentate bite of the ligand. The C—C N fragment is essentially linear at 179.5 (9)° and the C N bond length of 1.125 (11) Å indicates predominantly triple-bond character. In the crystal packing, there are no hydrogen-bonding or aurophilic inter­actions between the mol­ecules.

## Related literature

For background to bis­(diphenyl­phosphane)methane, Ph_2_PCH_2_PPh_2_, (dppm), see: Puddephatt (1983 ▶); Minahan & Hill (1984 ▶). For polymorphs of the related complex [(AuCl)_2_(dppm)], see: Schmidbaur *et al.* (1977 ▶); Healy (2003 ▶). For use of the anionic version of the ligand used in the present study, see: Ruiz *et al.* (1996 ▶); Mosquera *et al.* (2001 ▶). For recent work on bis­(diphenyl­phosphane)acetonitrile, see: Braun *et al.* (2007 ▶); Spannhoff *et al.* (2009 ▶). For background to our inter­est in dinuclear gold(I) complexes, see: Van Zyl (2010 ▶).
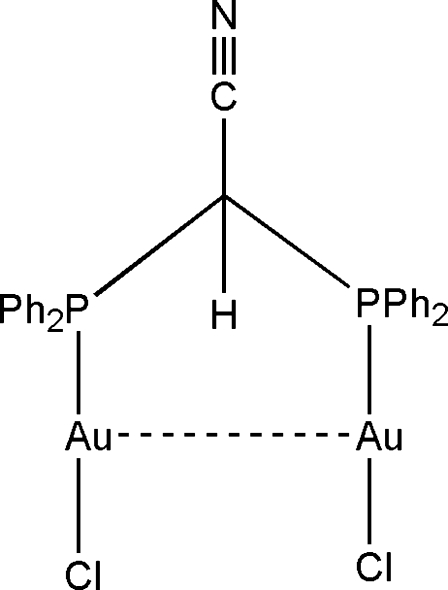


## Experimental

### 

#### Crystal data

[Au_2_Cl_2_(C_26_H_21_NP_2_)]
*M* *_r_* = 874.21
Orthorhombic, 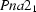

*a* = 13.9062 (8) Å
*b* = 12.6837 (7) Å
*c* = 14.7938 (8) Å
*V* = 2609.4 (3) Å^3^
*Z* = 4
Mo *K*α radiation
μ = 11.58 mm^−1^
*T* = 173 K
0.22 × 0.21 × 0.19 mm

#### Data collection

Bruker APEXII CCD diffractometer
Absorption correction: multi-scan (*SADABS*; Bruker, 2009 ▶) *T*_min_ = 0.189, *T*_max_ = 0.216
35892 measured reflections
4770 independent reflections
4621 reflections with *I* > 2σ(*I*)
*R*_int_ = 0.059

#### Refinement

*R*[*F* ^2^ > 2σ(*F* ^2^)] = 0.025
*wR*(*F* ^2^) = 0.063
*S* = 1.05
4770 reflections
298 parameters
1 restraint
H-atom parameters constrained
Δρ_max_ = 0.76 e Å^−3^
Δρ_min_ = −0.81 e Å^−3^
Absolute structure: Flack (1983 ▶), 2283 Friedel pairs
Flack parameter: 0.014 (8)

### 

Data collection: *COSMO* (Bruker, 2009 ▶); cell refinement: *APEX2* (Bruker, 2009 ▶); data reduction: *SAINT*; program(s) used to solve structure: *SHELXS97* (Sheldrick, 2008 ▶); program(s) used to refine structure: *SHELXL97* (Sheldrick, 2008 ▶); molecular graphics: *SHELXTL* (Sheldrick, 2008 ▶); software used to prepare material for publication: *SHELXTL*.

## Supplementary Material

Crystal structure: contains datablocks global, I. DOI: 10.1107/S1600536810049834/fj2370sup1.cif

Structure factors: contains datablocks I. DOI: 10.1107/S1600536810049834/fj2370Isup2.hkl

Additional supplementary materials:  crystallographic information; 3D view; checkCIF report

## Figures and Tables

**Fig. 1.**
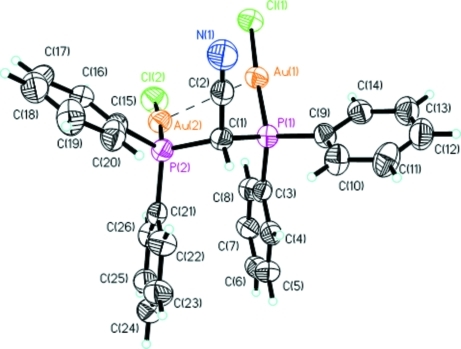
Molecular structure of the title complex showing the atom numbering scheme. Ellipsoids are drawn at the 50% probability level.

## supplementary crystallographic information

### Comment

Interest in the chemistry of the ligand bis(diphenylphosphane)acetonitrile, (dppm-CN), was recently rejuvenated with the facile preparation thereof starting with readily available acetonitrile (Braun *et al.*, 2007). Development of its chemistry followed thereafter, including the observed sharp increase in acidity by the replacement of a proton with a cyano group on the bridging carbon atom of the related ligand dppm (Spannhoff *et al.*, 2009, and references therein). We prepared dppm-CN through slight modification of the stoichiometric amounts used in the literature procedure (Braun *et al.*, 2007). Acetonitrile was doubly deprotonated with *n-*BuLi (2 molar equivalents) to yield the proposed intermediate Li_2_[CHCN] (not isolated), which was treated *in situ* with Ph_2_PCl (2 molar equivalents) to form dppm-CN. In continuing our interest in dinuclear gold(I) complexes (Van Zyl, 2010), we report the first structural investigation of a gold(I) complex with the ligand dppm-CN. Complex (I) was formed in CH_2_Cl_2_ solution from the reaction between the ligand and [AuCl(tht)] (tht = tetrahydrothiophene) (molar ratio 1:2) (see Experimental). The solution ^31^P NMR spectrum of complex(I) showed a singlet peak resonating at δ = 34.8 p.p.m. for the two equivalent P atoms. Complex (I) can be compared with the related [(AuCl)_2_dppm] complex which exists as two polymorphs. The first polymorph (monoclinic) (Schmidbaur *et al.*, 1977) contains an intramolecular Au···Au interaction of 3.351 (2) Å with no intermolecular Au···Au interaction, whilst the second polymorph (triclinic) (Healy, 2003) contains neither intra- (5.617 (3) Å) nor intermolecular Au···Au interactions. The cause for the structural difference between the two polymorphs can be found in two different conformational structures of the dppm ligand.

### Experimental

Preparation and characterization of complex(I): A solution of [AuCl(tht)] (156 mg, 0.48 mmol) in dichloromethane (10 ml) was slowly added to a solution of dppm-CN (100 mg, 0.24 mmol) in dichloromethane (5 ml) and the mixture stirred for 45 minutes at room temperature. All solvent and tht were then removed under reduced pressure to give complex (I). Dry Et_2_O (3 *x* 2 ml) was used to wash the product which was then further dried *in vacuo* overnight. The product was obtained as a free-flowing off-white powder. Yield (185 mg, 0.21 mmol) 88%. Mp: 155–158 °C; Elemental analysis for complex C_26_H_21_Au_2_Cl_2_NP_2_ Found: C, 35.21; H, 2.28 requires C, 35.72; H, 2.42%. ^1^H NMR (400 MHz, CDCl_3_, 298 K) δ_H_= 7.68–7.53 (m, 20H, Ph); 5.52 (t, 1H; CH, ^2^J_P,H_ = 12.77 Hz). ^31^P NMR (101 MHz, CDCl_3_, 298 K) δ_P _= 34.8 (s, 2P). IR (KBr, cm^-1^): 2243 ν(CN). ESI-MS: m/*z* 875 [*M*+]. Single crystals were obtained by slow diffusion of dry hexane into a saturated solution of dry dichloromethane.

### Refinement

All non-hydrogen atoms are refined anisotropically. H atoms were calculated by geometrical methods and refined as a riding model. The Flack parameter (Flack, 1983) is used to determine chirality of the crystal studied, the value should be near zero, a value of one is the other enantiomer and a value of 0.5 is racemic. The Flack parameter was refined to 0.014 (8), confirming the absolute stereochemistry.

### Crystal data

**Table:**

[Au_2_Cl_2_(C_26_H_21_NP_2_)]	*F*(000) = 1624
*M**_r_* = 874.21	*D*_x_ = 2.225 Mg m^−^^3^
Orthorhombic, *P**n**a*2_1_	Mo *K*α radiation, λ = 0.71073 Å
Hall symbol: P 2c -2n	Cell parameters from 9778 reflections
*a* = 13.9062 (8) Å	θ = 2.6–25.3°
*b* = 12.6837 (7) Å	µ = 11.58 mm^−^^1^
*c* = 14.7938 (8) Å	*T* = 173 K
*V* = 2609.4 (3) Å^3^	Chunk, colourless
*Z* = 4	0.22 × 0.21 × 0.19 mm

### Data collection

**Table:**

Bruker APEXII CCD diffractometer	4770 independent reflections
Radiation source: fine-focus sealed tube	4621 reflections with *I* > 2σ(*I*)
graphite	*R*_int_ = 0.059
Detector resolution: 836.6 pixels mm^-1^	θ_max_ = 25.4°, θ_min_ = 2.1°
ω and φ 0.5° scans	*h* = −16→16
Absorption correction: multi-scan (*SADABS*; Bruker, 2009)	*k* = −15→15
*T*_min_ = 0.189, *T*_max_ = 0.216	*l* = −17→17
35892 measured reflections	

### Refinement

**Table:**

Refinement on *F*^2^	Secondary atom site location: difference Fourier map
Least-squares matrix: full	Hydrogen site location: inferred from neighbouring sites
*R*[*F*^2^ > 2σ(*F*^2^)] = 0.025	H-atom parameters constrained
*wR*(*F*^2^) = 0.063	*w* = 1/[σ^2^(*F*_o_^2^) + (0.011*P*)^2^ + 3.9118*P*] where *P* = (*F*_o_^2^ + 2*F*_c_^2^)/3
*S* = 1.05	(Δ/σ)_max_ = 0.001
4770 reflections	Δρ_max_ = 0.76 e Å^−^^3^
298 parameters	Δρ_min_ = −0.81 e Å^−^^3^
1 restraint	Absolute structure: Flack (1983), 2283 Friedel pairs
Primary atom site location: structure-invariant direct methods	Flack parameter: 0.014 (8)

### Special details

**Table:**

Experimental. Data was collected using a BRUKER CCD (charge coupled device) based diffractometer equipped with an Oxford low-temperature apparatus operating at 173 K. A suitable crystal was chosen and mounted on a glass fiber or nylon loop using Paratone oil for Mo radiation and Mineral oil for Copper radiation. Data were measured using omega and phi scans of 0.5° per frame for 30 s. The total number of images were based on results from the program COSMO where redundancy was expected to be 4 and completeness to 0.83Å to 100%. Cell parameters were retrieved using *APEX* II software and refined using *SAINT* on all observed reflections.Data reduction was performed using the *SAINT* software which corrects for Lp. Scaling and absorption corrections were applied using *SADABS6* multi-scan technique, supplied by George Sheldrick. The structures are solved by the direct method using the *SHELXS97* program and refined by least squares method on F2, *SHELXL97*, incorporated in *SHELXTL*.All H atoms were placed in calculated positions and refined using a riding model. C—H(aromatic) = 0.94 Å and *U*_iso_(H) = 1.2Ueq(C) C—H (alaphatic) = 0.99 Å and *U*_iso_(H) = 1.2Ueq(C) CH2 = 0.98 Å and *U*_iso_(H) = 1.2Ueq(C) CH3 = 0.97Å and *U*_iso_(H) = 1.5Ueq(C) N—H = 0.86 (0.92)Å and *U*_iso_(H) = 1.2 *U*_eq_(N) O—H(alcohol) = 0.85Åand *U*_iso_(H) = 1.2Ueq(O) O—H(acid) = 0.82 Å and *U*_iso_(H) = 1.5Ueq(O)
Geometry. All e.s.d.'s (except the e.s.d. in the dihedral angle between two l.s. planes) are estimated using the full covariance matrix. The cell e.s.d.'s are taken into account individually in the estimation of e.s.d.'s in distances, angles and torsion angles; correlations between e.s.d.'s in cell parameters are only used when they are defined by crystal symmetry. An approximate (isotropic) treatment of cell e.s.d.'s is used for estimating e.s.d.'s involving l.s. planes.
Refinement. Refinement of *F*^2^ against ALL reflections. The weighted *R*-factor *wR* and goodness of fit *S* are based on *F*^2^, conventional *R*-factors *R* are based on *F*, with *F* set to zero for negative *F*^2^. The threshold expression of *F*^2^ > σ(*F*^2^) is used only for calculating *R*-factors(gt) *etc*. and is not relevant to the choice of reflections for refinement. *R*-factors based on *F*^2^ are statistically about twice as large as those based on *F*, and *R*- factors based on ALL data will be even larger.

### Fractional atomic coordinates and isotropic or equivalent isotropic displacement parameters (Å^2^)

**Table:**

	*x*	*y*	*z*	*U*_iso_*/*U*_eq_	
Cl1	−0.07615 (15)	0.82567 (17)	0.92236 (14)	0.0565 (5)	
Au1	−0.059247 (18)	0.86691 (2)	0.77316 (2)	0.04850 (8)	
Au2	0.148444 (19)	0.78078 (2)	0.72563 (2)	0.05055 (8)	
C4	−0.0452 (5)	0.8105 (7)	0.4587 (5)	0.0498 (17)	
H4	−0.0476	0.8790	0.4328	0.060*	
Cl2	0.13552 (17)	0.64675 (18)	0.82822 (17)	0.0687 (5)	
P2	0.16917 (13)	0.90358 (15)	0.61806 (13)	0.0437 (4)	
P1	−0.05098 (13)	0.91073 (16)	0.62748 (13)	0.0445 (4)	
C5	−0.0428 (6)	0.7239 (8)	0.4036 (6)	0.063 (2)	
H5	−0.0420	0.7325	0.3398	0.076*	
C6	−0.0414 (6)	0.6240 (7)	0.4409 (6)	0.057 (2)	
H6	−0.0424	0.5640	0.4025	0.069*	
C7	−0.0388 (5)	0.6106 (6)	0.5327 (6)	0.0510 (17)	
H7	−0.0357	0.5417	0.5577	0.061*	
C8	−0.0406 (5)	0.6990 (6)	0.5898 (6)	0.0501 (17)	
H8	−0.0394	0.6905	0.6536	0.060*	
C3	−0.0442 (5)	0.7994 (5)	0.5514 (5)	0.0408 (14)	
C15	0.2655 (5)	0.9971 (6)	0.6416 (5)	0.0490 (17)	
C16	0.3248 (5)	0.9752 (7)	0.7136 (5)	0.0539 (18)	
H16	0.3108	0.9175	0.7523	0.065*	
C17	0.4056 (6)	1.0377 (7)	0.7298 (7)	0.070 (2)	
H17	0.4486	1.0205	0.7775	0.083*	
C19	0.3635 (6)	1.1447 (7)	0.6058 (8)	0.068 (3)	
H19	0.3775	1.2027	0.5674	0.082*	
C20	0.2838 (5)	1.0835 (6)	0.5887 (6)	0.0572 (19)	
H20	0.2416	1.1012	0.5405	0.069*	
C1	0.0603 (5)	0.9886 (5)	0.6014 (5)	0.0449 (16)	
H1	0.0579	1.0120	0.5370	0.054*	
C9	−0.1500 (5)	0.9924 (6)	0.5901 (5)	0.0480 (17)	
C10	−0.1467 (5)	1.0590 (7)	0.5165 (6)	0.058 (2)	
H10	−0.0883	1.0675	0.4839	0.069*	
C11	−0.2286 (6)	1.1138 (6)	0.4897 (7)	0.065 (2)	
H11	−0.2268	1.1594	0.4388	0.078*	
C14	−0.2361 (5)	0.9810 (5)	0.6381 (5)	0.0496 (17)	
H14	−0.2393	0.9350	0.6887	0.059*	
C2	0.0620 (5)	1.0809 (7)	0.6594 (6)	0.057 (2)	
N1	0.0639 (6)	1.1521 (7)	0.7045 (7)	0.086 (3)	
C21	0.1903 (5)	0.8482 (6)	0.5076 (5)	0.0443 (15)	
C22	0.1955 (6)	0.9097 (6)	0.4297 (5)	0.0536 (18)	
H22	0.1890	0.9842	0.4333	0.064*	
C23	0.2103 (6)	0.8614 (8)	0.3471 (6)	0.064 (2)	
H23	0.2137	0.9033	0.2940	0.077*	
C25	0.2138 (6)	0.6929 (7)	0.4167 (6)	0.0544 (18)	
H25	0.2198	0.6185	0.4122	0.065*	
C26	0.1985 (5)	0.7393 (5)	0.4999 (5)	0.0477 (17)	
H26	0.1935	0.6964	0.5523	0.057*	
C18	0.4224 (6)	1.1235 (7)	0.6765 (8)	0.070 (3)	
H18	0.4754	1.1684	0.6890	0.085*	
C24	0.2203 (6)	0.7542 (7)	0.3403 (6)	0.0578 (19)	
H24	0.2317	0.7224	0.2832	0.069*	
C13	−0.3161 (6)	1.0369 (7)	0.6115 (6)	0.0594 (19)	
H13	−0.3741	1.0303	0.6450	0.071*	
C12	−0.3136 (6)	1.1007 (7)	0.5390 (7)	0.067 (2)	
H12	−0.3701	1.1372	0.5211	0.080*	

### Atomic displacement parameters (Å^2^)

**Table:**

	*U*^11^	*U*^22^	*U*^33^	*U*^12^	*U*^13^	*U*^23^
Cl1	0.0629 (12)	0.0548 (11)	0.0517 (11)	0.0020 (9)	0.0055 (9)	−0.0002 (9)
Au1	0.04863 (14)	0.05302 (15)	0.04384 (14)	0.00237 (11)	0.00289 (15)	−0.00088 (14)
Au2	0.05242 (15)	0.05362 (15)	0.04561 (14)	0.00443 (12)	−0.00177 (14)	0.00171 (15)
C4	0.041 (4)	0.059 (4)	0.050 (4)	0.006 (3)	0.003 (3)	0.002 (3)
Cl2	0.0773 (14)	0.0705 (13)	0.0582 (12)	0.0084 (11)	0.0039 (11)	0.0115 (10)
P2	0.0410 (9)	0.0456 (10)	0.0444 (9)	0.0022 (7)	−0.0042 (8)	−0.0037 (8)
P1	0.0394 (9)	0.0489 (10)	0.0453 (10)	0.0034 (7)	−0.0022 (7)	−0.0012 (8)
C5	0.056 (5)	0.093 (7)	0.041 (4)	0.004 (4)	0.002 (4)	−0.006 (4)
C6	0.046 (4)	0.063 (5)	0.063 (5)	−0.009 (4)	0.009 (4)	−0.020 (4)
C7	0.049 (4)	0.048 (4)	0.056 (5)	−0.007 (3)	0.009 (4)	−0.009 (3)
C8	0.049 (4)	0.047 (4)	0.055 (4)	−0.014 (3)	0.004 (3)	−0.003 (3)
C3	0.034 (3)	0.047 (4)	0.041 (4)	−0.002 (3)	−0.004 (3)	0.001 (3)
C15	0.040 (3)	0.052 (4)	0.055 (4)	0.005 (3)	−0.006 (3)	−0.016 (3)
C16	0.045 (4)	0.071 (5)	0.046 (4)	0.003 (3)	0.001 (3)	−0.016 (4)
C17	0.059 (4)	0.084 (6)	0.066 (5)	0.002 (4)	−0.018 (5)	−0.019 (5)
C19	0.055 (5)	0.044 (4)	0.106 (8)	−0.002 (3)	−0.003 (5)	−0.005 (4)
C20	0.047 (4)	0.046 (4)	0.078 (5)	0.001 (3)	−0.008 (4)	−0.005 (4)
C1	0.044 (4)	0.045 (4)	0.046 (4)	0.005 (3)	−0.005 (3)	−0.007 (3)
C9	0.043 (4)	0.047 (4)	0.054 (4)	0.008 (3)	−0.002 (3)	−0.005 (3)
C10	0.046 (4)	0.061 (5)	0.066 (5)	−0.001 (3)	−0.002 (4)	0.012 (4)
C11	0.063 (5)	0.050 (5)	0.082 (6)	−0.001 (4)	−0.021 (5)	0.012 (4)
C14	0.051 (4)	0.040 (4)	0.058 (4)	−0.002 (3)	0.001 (3)	−0.007 (3)
C2	0.042 (4)	0.063 (5)	0.067 (5)	0.005 (3)	−0.008 (4)	−0.017 (4)
N1	0.072 (5)	0.071 (5)	0.115 (8)	0.014 (4)	−0.015 (5)	−0.045 (5)
C21	0.033 (3)	0.052 (4)	0.048 (4)	−0.001 (3)	−0.004 (3)	−0.006 (3)
C22	0.063 (5)	0.045 (4)	0.052 (4)	0.000 (4)	0.005 (4)	0.001 (3)
C23	0.066 (5)	0.076 (6)	0.051 (5)	0.002 (4)	0.007 (4)	0.007 (4)
C25	0.049 (4)	0.053 (4)	0.061 (5)	0.001 (3)	−0.003 (4)	−0.012 (4)
C26	0.043 (4)	0.045 (4)	0.055 (4)	−0.001 (3)	−0.007 (3)	0.003 (3)
C18	0.052 (5)	0.059 (5)	0.100 (7)	−0.002 (4)	0.002 (5)	−0.031 (5)
C24	0.051 (4)	0.072 (5)	0.051 (5)	−0.004 (4)	0.005 (4)	−0.006 (4)
C13	0.046 (4)	0.062 (5)	0.071 (5)	0.000 (4)	−0.003 (4)	−0.003 (4)
C12	0.053 (5)	0.066 (5)	0.082 (6)	0.011 (4)	−0.014 (5)	−0.004 (5)

### Geometric parameters (Å, °)

**Table:**

Cl1—Au1	2.281 (2)	C19—C20	1.377 (11)
Au1—P1	2.229 (2)	C19—H19	0.9500
Au1—Au2	3.1669 (4)	C20—H20	0.9500
Au2—P2	2.245 (2)	C1—C2	1.451 (10)
Au2—Cl2	2.286 (2)	C1—H1	1.0000
C4—C5	1.367 (12)	C9—C10	1.379 (12)
C4—C3	1.379 (11)	C9—C14	1.398 (10)
C4—H4	0.9500	C10—C11	1.391 (11)
P2—C21	1.802 (7)	C10—H10	0.9500
P2—C15	1.822 (7)	C11—C12	1.399 (13)
P2—C1	1.874 (7)	C11—H11	0.9500
P1—C3	1.808 (7)	C14—C13	1.377 (11)
P1—C9	1.810 (7)	C14—H14	0.9500
P1—C1	1.876 (7)	C2—N1	1.125 (11)
C5—C6	1.383 (13)	C21—C26	1.391 (10)
C5—H5	0.9500	C21—C22	1.394 (11)
C6—C7	1.369 (13)	C22—C23	1.383 (12)
C6—H6	0.9500	C22—H22	0.9500
C7—C8	1.404 (10)	C23—C24	1.370 (12)
C7—H7	0.9500	C23—H23	0.9500
C8—C3	1.396 (10)	C25—C24	1.374 (12)
C8—H8	0.9500	C25—C26	1.381 (11)
C15—C20	1.371 (11)	C25—H25	0.9500
C15—C16	1.375 (10)	C26—H26	0.9500
C16—C17	1.396 (11)	C18—H18	0.9500
C16—H16	0.9500	C24—H24	0.9500
C17—C18	1.364 (14)	C13—C12	1.344 (13)
C17—H17	0.9500	C13—H13	0.9500
C19—C18	1.355 (15)	C12—H12	0.9500
			
P1—Au1—Cl1	176.84 (7)	C15—C20—H20	120.1
P1—Au1—Au2	79.88 (5)	C19—C20—H20	120.1
Cl1—Au1—Au2	103.28 (5)	C2—C1—P2	112.0 (5)
P2—Au2—Cl2	175.21 (8)	C2—C1—P1	108.4 (5)
P2—Au2—Au1	92.01 (5)	P2—C1—P1	109.7 (4)
Cl2—Au2—Au1	92.15 (6)	C2—C1—H1	108.9
C5—C4—C3	120.7 (8)	P2—C1—H1	108.9
C5—C4—H4	119.6	P1—C1—H1	108.9
C3—C4—H4	119.6	C10—C9—C14	119.5 (6)
C21—P2—C15	107.9 (3)	C10—C9—P1	124.5 (6)
C21—P2—C1	103.7 (3)	C14—C9—P1	115.9 (6)
C15—P2—C1	104.1 (3)	C9—C10—C11	120.2 (8)
C21—P2—Au2	113.2 (3)	C9—C10—H10	119.9
C15—P2—Au2	114.2 (3)	C11—C10—H10	119.9
C1—P2—Au2	112.8 (3)	C10—C11—C12	118.9 (8)
C3—P1—C9	107.2 (3)	C10—C11—H11	120.5
C3—P1—C1	103.9 (3)	C12—C11—H11	120.5
C9—P1—C1	105.3 (3)	C13—C14—C9	119.6 (7)
C3—P1—Au1	114.2 (2)	C13—C14—H14	120.2
C9—P1—Au1	113.5 (3)	C9—C14—H14	120.2
C1—P1—Au1	111.9 (2)	N1—C2—C1	179.5 (9)
C4—C5—C6	119.9 (8)	C26—C21—C22	118.9 (7)
C4—C5—H5	120.0	C26—C21—P2	118.4 (6)
C6—C5—H5	120.0	C22—C21—P2	122.7 (6)
C7—C6—C5	120.7 (8)	C23—C22—C21	119.4 (8)
C7—C6—H6	119.7	C23—C22—H22	120.3
C5—C6—H6	119.7	C21—C22—H22	120.3
C6—C7—C8	119.8 (8)	C24—C23—C22	121.3 (8)
C6—C7—H7	120.1	C24—C23—H23	119.3
C8—C7—H7	120.1	C22—C23—H23	119.3
C3—C8—C7	119.0 (8)	C24—C25—C26	120.1 (8)
C3—C8—H8	120.5	C24—C25—H25	120.0
C7—C8—H8	120.5	C26—C25—H25	120.0
C4—C3—C8	119.9 (7)	C25—C26—C21	120.6 (7)
C4—C3—P1	122.6 (6)	C25—C26—H26	119.7
C8—C3—P1	117.5 (6)	C21—C26—H26	119.7
C20—C15—C16	119.4 (7)	C19—C18—C17	120.1 (8)
C20—C15—P2	123.2 (6)	C19—C18—H18	120.0
C16—C15—P2	117.3 (6)	C17—C18—H18	120.0
C15—C16—C17	120.2 (8)	C23—C24—C25	119.6 (8)
C15—C16—H16	119.9	C23—C24—H24	120.2
C17—C16—H16	119.9	C25—C24—H24	120.2
C18—C17—C16	119.4 (9)	C12—C13—C14	121.1 (8)
C18—C17—H17	120.3	C12—C13—H13	119.5
C16—C17—H17	120.3	C14—C13—H13	119.5
C18—C19—C20	121.1 (10)	C13—C12—C11	120.7 (8)
C18—C19—H19	119.4	C13—C12—H12	119.7
C20—C19—H19	119.4	C11—C12—H12	119.7
C15—C20—C19	119.7 (8)		
			
P1—Au1—Au2—P2	29.20 (7)	C21—P2—C1—P1	92.2 (4)
Cl1—Au1—Au2—P2	−150.94 (7)	C15—P2—C1—P1	−155.0 (4)
P1—Au1—Au2—Cl2	−148.43 (8)	Au2—P2—C1—P1	−30.6 (4)
Cl1—Au1—Au2—Cl2	31.43 (9)	C3—P1—C1—C2	179.9 (5)
Au1—Au2—P2—C21	−120.8 (2)	C9—P1—C1—C2	67.3 (6)
Au1—Au2—P2—C15	115.3 (3)	Au1—P1—C1—C2	−56.4 (6)
Au1—Au2—P2—C1	−3.4 (2)	C3—P1—C1—P2	−57.6 (4)
Au2—Au1—P1—C3	64.8 (2)	C9—P1—C1—P2	−170.2 (4)
Au2—Au1—P1—C9	−171.9 (3)	Au1—P1—C1—P2	66.1 (4)
Au2—Au1—P1—C1	−52.9 (2)	C3—P1—C9—C10	−76.6 (8)
C3—C4—C5—C6	1.4 (12)	C1—P1—C9—C10	33.7 (8)
C4—C5—C6—C7	−2.5 (13)	Au1—P1—C9—C10	156.3 (6)
C5—C6—C7—C8	2.1 (12)	C3—P1—C9—C14	100.1 (6)
C6—C7—C8—C3	−0.6 (11)	C1—P1—C9—C14	−149.7 (5)
C5—C4—C3—C8	0.1 (11)	Au1—P1—C9—C14	−27.0 (6)
C5—C4—C3—P1	−178.1 (6)	C14—C9—C10—C11	−0.5 (12)
C7—C8—C3—C4	−0.5 (10)	P1—C9—C10—C11	176.0 (7)
C7—C8—C3—P1	177.8 (5)	C9—C10—C11—C12	0.5 (14)
C9—P1—C3—C4	48.9 (7)	C10—C9—C14—C13	−0.4 (11)
C1—P1—C3—C4	−62.3 (6)	P1—C9—C14—C13	−177.2 (6)
Au1—P1—C3—C4	175.5 (5)	C15—P2—C21—C26	122.9 (6)
C9—P1—C3—C8	−129.4 (6)	C1—P2—C21—C26	−127.0 (6)
C1—P1—C3—C8	119.4 (6)	Au2—P2—C21—C26	−4.4 (6)
Au1—P1—C3—C8	−2.7 (6)	C15—P2—C21—C22	−59.2 (7)
C21—P2—C15—C20	59.5 (7)	C1—P2—C21—C22	50.9 (7)
C1—P2—C15—C20	−50.3 (7)	Au2—P2—C21—C22	173.5 (6)
Au2—P2—C15—C20	−173.8 (6)	C26—C21—C22—C23	−1.1 (12)
C21—P2—C15—C16	−117.1 (6)	P2—C21—C22—C23	−179.0 (7)
C1—P2—C15—C16	133.2 (6)	C21—C22—C23—C24	−0.2 (13)
Au2—P2—C15—C16	9.7 (6)	C24—C25—C26—C21	−0.5 (12)
C20—C15—C16—C17	−3.1 (11)	C22—C21—C26—C25	1.5 (11)
P2—C15—C16—C17	173.5 (6)	P2—C21—C26—C25	179.4 (6)
C15—C16—C17—C18	3.3 (12)	C20—C19—C18—C17	2.9 (15)
C16—C15—C20—C19	2.8 (12)	C16—C17—C18—C19	−3.2 (14)
P2—C15—C20—C19	−173.7 (7)	C22—C23—C24—C25	1.2 (14)
C18—C19—C20—C15	−2.7 (14)	C26—C25—C24—C23	−0.8 (13)
C21—P2—C1—C2	−147.4 (6)	C9—C14—C13—C12	1.4 (12)
C15—P2—C1—C2	−34.5 (7)	C14—C13—C12—C11	−1.5 (14)
Au2—P2—C1—C2	89.8 (6)	C10—C11—C12—C13	0.5 (14)
